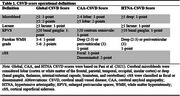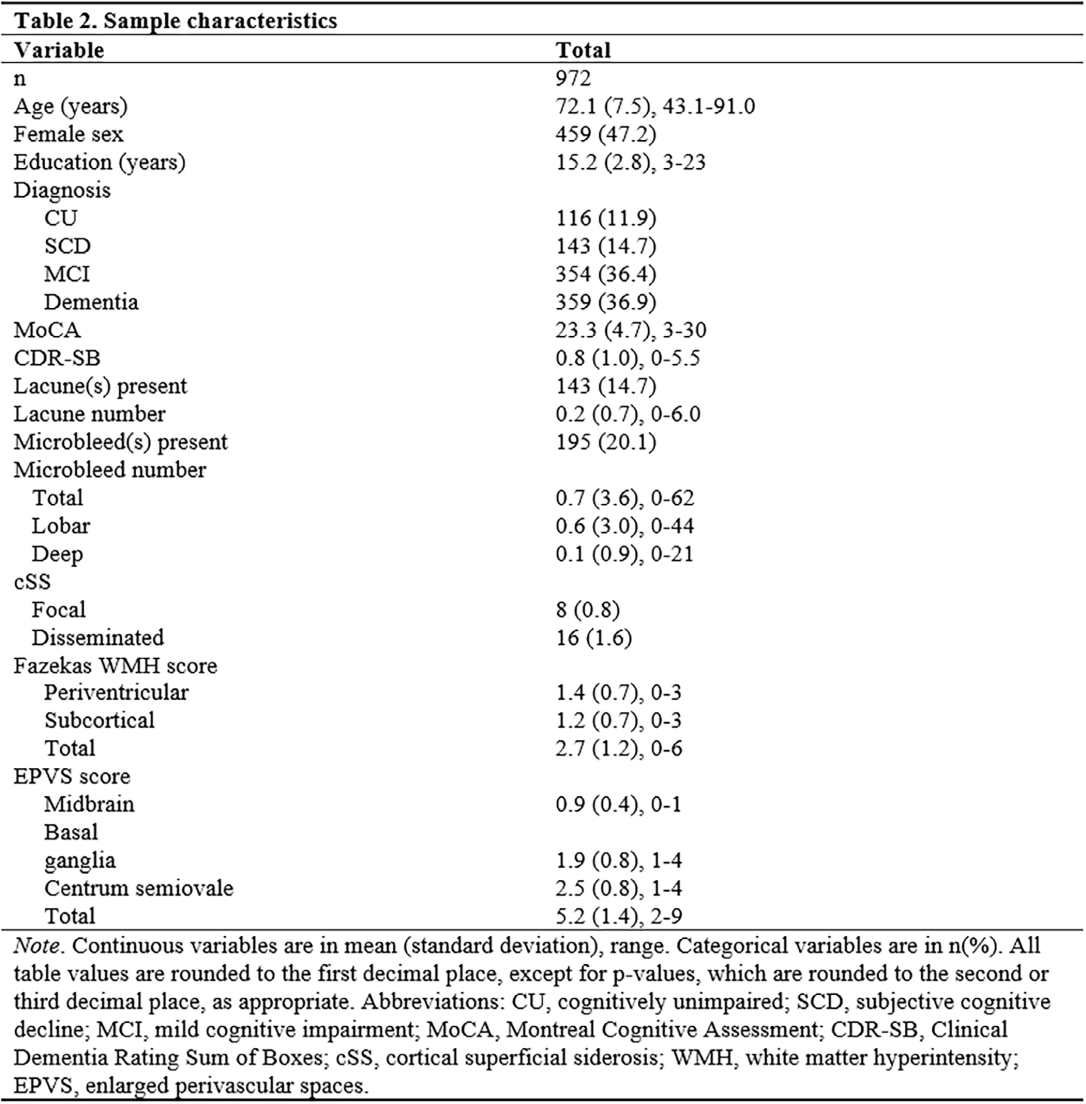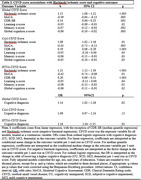# Association of imaging markers and overall burden of cerebral small vessel disease with cognition in COMPASS‐ND

**DOI:** 10.1002/alz70856_104717

**Published:** 2026-01-07

**Authors:** Dylan X. Guan, Zahinoor Ismail, Graham A. McLeod, Shahab Marzoughi, Eric E. Smith, Aravind Ganesh

**Affiliations:** ^1^ Hotchkiss Brain Institute, University of Calgary, Calgary, AB, Canada; ^2^ University of Calgary, Calgary, AB, Canada

## Abstract

**Background:**

Cerebral small vessel disease (CSVD) is the most common cause of vascular cognitive impairment, but its relationship to cognition across the neurocognitive spectrum is not fully understood. CSVD burden can be inferred from several magnetic resonance imaging (MRI) markers, which can be combined to generate a CSVD score. We investigated the association between CSVD score with various cognitive measures.

**Method:**

Baseline data from 972 participants [Table 1] from the Comprehensive Assessment of Neurodegeneration and Dementia (COMPASS‐ND) study were analyzed [11.9% cognitively unimpaired [CU], 14.7% subjective cognitive decline [SCD], 36.4% mild cognitive impairment [MCI], 36.9% dementia). Brain MRI scans were visually rated for Standards for Reporting Vascular Changes on Neuroimaging (STRIVE)‐based evidence of vascular brain injury (lacunes, microbleeds, white matter hyperintensities [WMH], cortical superficial siderosis [cSS], enlarged perivascular spaces [EPVS]). Three CSVD scores corresponding to global, cerebral amyloid angiopathy (CAA‐CSVD)‐specific, and hypertensive arteriopathy (HTNA‐CSVD)‐specific CSVD burden were generated [Table 2]. Cognitive measures included the Montreal Cognitive Assessment (MoCA), Clinical Dementia Rating sum of boxes (CDR‐SB), and a composite neuropsychological battery test z‐score. We modelled CSVD score (exposure) associations with five outcomes: Hachinski ischemic score (negative binomial regression), MoCA total score (linear regression), CDR‐SB (median quantile regression), and neuropsychological battery composite z‐score (linear regression), cognitive diagnosis (ordinal logistic regression). Covariates included age, sex, and education.

**Result:**

Global, CAA‐ and HTNA‐CSVD scores were all associated with greater Hachinski ischemic score, poorer MoCA score, higher CDR‐SB, and poorer composite neuropsychological battery test z‐score [Table 3]. Both global CSVD score (adjusted odds ratio [aOR]=1.14, 95%CI: [1.02, 1.26], *p* = .02) and HTNA‐CSVD score (aOR=1.18, 95%CI: [1.04, 1.35], *p* = .01) were associated with higher odds of a more severe cognitive diagnosis compared to a less severe cognitive diagnosis (e.g., dementia vs MCI/SCD/CU), but not CAA‐CSVD score (aOR=1.09, 95%CI: [0.97, 1.24], *p* = .15).

**Conclusion:**

Older adults with greater CSVD burden, as evidenced by multiple MRI markers, exhibit poorer cognition and functional performance across the neurocognitive continuum. Notably, these associations were observed not only for global CSVD but also for both CAA‐ and HTNA‐specific CSVD scores, suggesting the importance of evaluating these specific pathologies when assessing cerebrovascular contributions to cognitive decline.